# Reward Modulates Affective Priming Effect in Cognitive Conflict Processing: Electrophysiological Evidence

**DOI:** 10.3389/fnhum.2020.00059

**Published:** 2020-02-26

**Authors:** Fada Pan, Yuhong Ou, Xinni Zhang

**Affiliations:** School of Education Science, Nantong University, Nantong, China

**Keywords:** event-related potentials, cognitive control, cognitive conflict, Flanker task, reward

## Abstract

Previous research demonstrated that cognitive conflict could induce an affective priming effect, and the stage (detection/resolution) of conflict processing led to different directions (positive/negative) of the affective priming effect. We suggested that rewards play a critical role in the affective priming effect on conflict resolution. The present study used event-related potentials (ERPs), using the arrow flanker task as primes and choosing specific affective words as targets to investigate the affective priming effect induced by cognitive conflict during the resolution stage. Our question was whether rewards created a modulating effect. Participants were asked to judge the congruency of the prime stimuli and then evaluate the valence of the target words. For behavioral results, the conflict effect was significant, and the reward promoted the behavioral performance of participants. For ERP results, enhanced N2 amplitudes for incongruent primes indicated a significant conflict effect. More importantly, as expected, in the rewarded condition, the enhanced N400 amplitudes for positive targets following incongruent primes were found, indicating a positive priming effect. However, in the unrewarded condition, the reduced N400 amplitudes for positive targets following incongruent primes were found, indicating conflict resolution hindered the processing of positive stimuli. These findings suggested that cognitive conflict-induced the positive priming effect during the resolution stage and that rewards had a moderating effect on the positive priming effect.

## Introduction

Cognitive control refers to the ability to focus on goal-directed information and ignore goal-interfered information (Botvinick et al., 2001). If the goal-directed information is inconsistent with the goal-interfered information, the cognitive conflict is induced. The Stroop (Stroop, 1935) and Flanker paradigm (Eriksen and Eriksen, 1974) are typical congruency tasks designed to induce cognitive conflict in the laboratory. A Flanker paradigm single-trial usually consists of two components: the target and the distracter. The Flanker task contains two congruent stimuli (<<<<<, > > > > >) and two incongruent stimuli (<<><<, > >< > >). If the target and the distracters do not match, the arrows on both sides (distracters) will hinder the response to the target. This is called the “conflict effect.”

Cognitive control was once considered “pure” cognition (Botvinick et al., 1999, 2001; MacDonald et al., 2000; Miller and Cohen, 2001; Kerns et al., 2004). Recently, studies of cognitive control have concluded that cognitive control integrates cognition and emotion. The conflict monitoring theory suggests that the anterior cingulate cortex (ACC) monitors for response conflicts and signals the need for additional cognitive control through the executive unit (dorsolateral prefrontal cortex, DLPFC), thereby activating cognitive control (Botvinick et al., 1999, 2001). In the present research, we focused on the affective nature of the ACC (Shackman et al., 2011; Chen et al., 2014; Braem et al., 2017). The outcome evaluation account theory (Botvinick, 2007) suggests that ACC monitors any negative performance outcomes and information that serves as an aversive learning signal for future action selection (Gehring and Willoughby, 2002; Holroyd and Coles, 2002; Nieuwenhuis et al., 2004). Reconciling the two theories above, Botvinick (2007) suggested that the ACC might not only monitor for conflicts but also monitor for aversive signals in general. As ACC functions were discovered, the complexity and diversity of the ACC function (Wager et al., 2016) brought about reinforcement learning models of ACC (Rushworth and Behrens, 2008; Silvetti et al., 2011, 2014, 2018; Shenhav et al., 2013, 2016; Vassena et al., 2014). The reward value and prediction model (RVPM; Silvetti et al., 2011, 2014) suggested that ACC could code for the value of stimuli or actions. More specifically, the ACC could code for the differences between such values and the actual reward (reward prediction error) which are used for updating value estimates. Notably, the RVPM estimates only the reward expectations. The expected value of control (EVC) theory suggested that ACC is a system of optimal allocations of control. The ACC integrates information from an expected payoff and the amount of control that must be invested to achieve that payoff, thereby licensing the associated cognitive effort (Shenhav et al., 2016).

Over the past few years, researchers have shown great interest in the affective nature of the cognitive conflict. These studies primarily utilized a combination of the congruency task and the affective priming task. The combination makes the affective priming task distinguishable from the traditional one. The prime component in the special paradigm is congruency task (e.g., the Flanker task), and the target component is well-defined affective stimuli (e.g., the word “death” or “love”), which are used to perceive the affective nature of primes. The combination one aimed to investigate the affective effect of cognitive conflict. That was, the affective nature of the cognitive conflict was negative or positive. If the valence (negative/positive) of congruency primes reduces RTs for the evaluation of subsequent negative targets (i.e., words with a negative meaning), the affective effect of congruency primes could be termed the “aversive signal effect.” If the valence (negative/positive) of congruency primes reduced RTs for the evaluation of subsequent positive targets (i.e., words with a positive meaning), the affective effect of congruency primes could be termed the “positive priming effect.” In general, the effect of congruency primes on the evaluation of target words through the affective priming paradigm was termed the “affective priming effect” (Dreisbach and Fischer, 2012b; Fritz and Dreisbach, 2013, 2015; Schouppe et al., 2015).

In the beginning, conflicts were found to be viewed as aversive signals. Dreisbach and Fischer (2012a) asked participants to evaluate the valence of affective words following congruent or incongruent Stroop primes. The results indicated that conflicts could be viewed as aversive signals. Later, they used neutral words as target words (Fritz and Dreisbach, 2013) and found that participants evaluated neutral words more negatively after incongruent primes than after congruent primes. Recently, Pan et al. (2019) used arrow Flanker as primes and tested individuals with low-trait and high-trait anxiety. They found that conflicts could be viewed as aversive signals, and high-trait anxiety could promote the negative effect.

However, some studies found that cognitive conflicts could also induce a positive priming effect. Schouppe et al. (2015) informed participants to respond to the Flanker task (Experiment 2A) or the Stroop task (Experiment 2B) first, and then evaluate the valence of affective words. In these two experiments, they found that once the conflict was successfully resolved, a positive priming effect would be induced. Moreover, the positive priming effect was more likely to appear after a correct response on incongruent trials than on congruent trials. Fritz and Dreisbach (2015) determined that the stimulus onset asynchrony (SOA) played a critical role in the affective priming effect of cognitive conflicts. Conflicts could be viewed as aversive signals when the SOA was 200 ms or 400 ms. However, when the SOA was 800 ms, a positive priming effect would be induced. Pan et al. (2016) found similar results to Fritz and Dreisbach (2015). Conflicts could be viewed as aversive signals at the stage of conflict detection (SOA = 200 ms), and a positive priming effect was induced at the stage of conflict resolution (SOA = 800 ms).

In conclusion, these studies showed that the affective priming effect has two directions: one perceived as negative and the other as positive. The stage (detection/resolution) of conflict processing might be the leading cause resulted in different directions (positive/negative) of the affective priming effect. According to the RVPM, as well as findings from previous studies (Braem et al., 2012; Inzlicht et al., 2015), we suggested that it was the intrinsic and potential reward of conflict resolution, rather than conflict itself, that typically induced the positive priming effect. In other words, the aversive signal effect was conflict-based, while the positive priming effect was reward-based.

To verify that the reward modulated the affective priming effect during the conflict resolution stage, we established a rewarded condition and an unrewarded condition. The arrow flanker task was used as primes, and we chose effective words as targets. Event-related potentials (ERPs) were used for time precision. N2 at the frontal-central area were often observed in conflict detection with more negative amplitude for the incongruent condition compared to congruent condition (Kanske and Kotz, 2010; Pan et al., 2019). N400, a crucial index that sensitive to semantic processing, was chosen for target words (Pan et al., 2016, 2019; Saunders et al., 2017; Pauligk et al., 2019). We predicted that cognitive conflict would induce the affective priming effect and that the rewards would modulate the priming effect. Hence, an interaction among the priming condition, the target valence, and the reward condition would be found for both behavioral results and ERP results.

## Materials and Methods

This study was approved by the Human Ethics Committee of Nantong University. Written informed consent was obtained from all participants. Everyone was given a small gift in return for participating.

### Participants

We recruited 34 students (17 males and 17 females) aged 19–27 years (*M* = 21.4, *SD* = 2.1) from Nantong University, China. All participants were right-handed, had no mental illness, color blindness or color weakness, and had a normal or corrected-to-normal vision. Two participants’ data were excluded due to an error rate of more than 20% and less than 30 trials completed (Huffmeijer et al., 2014).

### Materials

The primes were Flanker stimuli consisting of a horizontal array of five arrows in four different combinations (i.e., <<<<<, <<><<, > > > > >, > >< > >). The direction of the central arrow could either match (congruent prime) or mismatch (incongruent prime) the direction of the neighboring arrows. In order to ensure the occurrence of the cognitive conflict and amplify the conflict, we made several modifications to the procedure (Forster et al., 2011; Larson et al., 2014). Primarily, neighboring arrows were presented for 100 ms. Subsequently, the central arrow and the neighboring arrows were presented for an additional 200 ms. Moreover, we decreased the proportion of incongruent trials (incongruent trials: congruent trials = 5:6) since conflict would be greater if incongruent trials were less than congruent trials (Carter et al., 2000).

The target stimuli were 120 Chinese words chosen from the Chinese Affective Words System (CAWS; Wang et al., 2008), including 60 positive words and 60 negative words. Two characteristics of the words were controlled: valance and arousal. The difference in valance between positive and negative words was significant (*t*_(118)_ = 71.401, *p* < 0.001), and there was no significant difference in arousal (*t*_(118)_ = −0.019, *p* > 0.05). The probability of each target word presenting in different prime/reward combinations was the same, and the frequency of each target word presenting was counterbalanced among participants.

### Procedure

The experiment was run on an Asus Computer and programmed in E-Prime 2.0. The stimuli were presented centrally on the computer screen with a white background. The prime and target stimuli were black characters presented in a 44-point Times New Roman font. For reducing participants’ anxiety, an explanation of the ERP experiment was provided. Then, the participants entered the electroencephalogram (EEG) laboratory and sat about 80 cm away from the front of the computer screen. The viewing angle of the stimuli on the screen was 9.87° (vertically) × 6.58° (horizontally).

Our experiment included a practice block and a formal block. Twenty trials were completed to ensure that the participants were familiar with the task in the practice block. The formal block contained 440 trials. The target words used in the practice block were different from those in the formal block. First, a fixation cross was presented for 500~1,000 ms. Then, the flankers were presented for 100 ms; after that, the central target stimulus along with the flankers was presented for 200 ms. Next, a blank screen was presented until a participants’ response. The participants were instructed to determine whether the direction of the middle arrow matched the arrows on both sides using their left hand by the key “Q” and “W,” and the maximum RT was 1,000 ms.

If participants responded slower than 1,000 ms or incorrectly, a meaningless symbol (“*”) would be presented centrally on the screen for 500 ms. If participants responded correctly on the Flanker task, there would be a rewarded trial or an unrewarded trial. On rewarded trials, the participant was given feedback in the form of a “+1” presented centrally on the screen for 500 ms. On unrewarded trials, the reward symbol was replaced by a meaningless symbol (“*”) presented centrally on the screen for 500 ms. Participants were informed that they could gain extra points according to the “quality” of the response. If their response had high “quality, ” the “+1” would be presented, which meant they had scored an extra point. Except for a small gift, the participant with the best score would additionally receive a gift worth 100¥. During each 1-min rest period, participants were informed of the “quality” of their responses in the previous part. It was worth noting that the rewarded trials were arranged so that if participants responded correctly on the Flanker task, half of them were rewarded trials, while others were unrewarded trials, and all were randomized following the congruent and incongruent trials separately.

**Figure 1:**
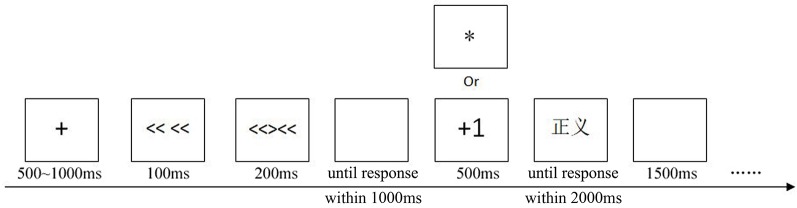
Scheme of a single trial used in the present study. “
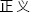
” means “justice” here.

Then, the target word appeared and remained on the screen until a response was made within 2,000 ms. The participants were told to respond to the valence of the word (positive or negative) using their right hand by the key “O” and “P.” The maximum RT was 2,000 ms. Finally, a blank screen was presented for 1,500 ms (Figure 1). The hands’ response to primes and targets has been counterbalanced. Participants could rest for 1 min every 110 trials. Moreover, the participants were told to focus their attention on the fixation cross and try their best not to swallow, frown, blink, etc.

### ERP Recordings and Data Pre-processing

EEG was recorded continuously using a 64 Ag-AgCl electrodes elastic cap placed according to the international 10-20 system, and the Neuroscan ERP workstation (Scan 4.5). REF and GND served as a reference electrode and the ground electrode on the top of the head and medial frontal aspect separately. The vertical electrooculogram (EOG) and horizontal EOG were placed above and below the participant’s left eye and outer canthi of both eyes. The sampling rate and bandpass were 1,000 Hz/channel and 0.05~100 Hz. EEG data and behavioral data were recorded simultaneously. Every participant washed his or her hair and dried the hair after washing in the laboratory first, and then we started to record until the impedances were maintained below 5KΩ with the conductive paste on the scalp.

EEGLAB (Version 11.0.0.0b), an open toolbox running under the MATLAB environment (Delorme and Makeig, 2004), was used for off-line analysis. We translated the average of M1 and M2 into a new reference. Then, the sampling rate was changed to 500 Hz/channel, and the bandpass was filtered to a range of 0.01~30 Hz. After that, eye blinks, eye movements, and other artifacts were removed from the averaging by the Independent Component Analysis (ICA). All epochs exceeding ±100 μV were automatically excluded from further processing. The acceptance rate of each participant was more than 80% of the trials with incorrect responses and artifacts excluded; additionally, more than 30 trials remained for each condition (Huffmeijer et al., 2014). Averages were computed separately for each condition and subject.

**Figure 2:**
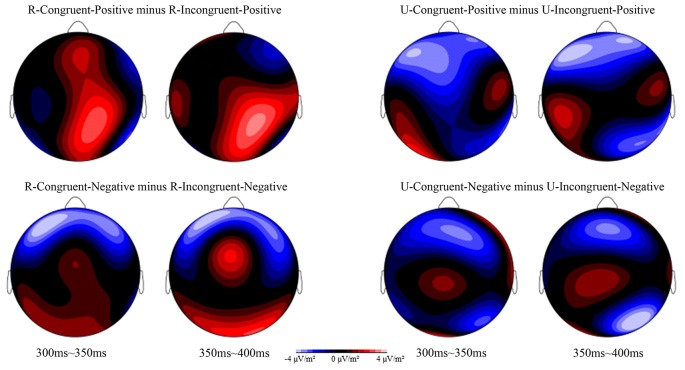
Topographic voltage map of mean amplitude differences for the affective priming effect at 300–350 ms and 350–400 ms in the four conditions: R-Congruent-Positive minus R-Incongruent-Positive, U-Congruent-Positive minus U-Incongruent-Positive, R-Congruent-Negative minus R-Incongruent-Negative, and U-Congruent-Negative minus U-Incongruent-Negative.

For the analysis of the conflict effect, according to previous research (Kanske and Kotz, 2010) and the topographic voltage map, we chose the N2 component with 5 electrode sites in the frontal areas: Fz, F3, F4, FCz, AF3, and AF4. The ERPs’ waveforms were time-locked to the onset of Flanker primes, and the average epoch was 1,000 ms. The EEG activity during the 200 ms prior to the onset of Flanker prime served as the baseline for the analysis of N2. The time window of N2 was 400 ms~500 ms. For the analysis of the affective priming effect, according to previous studies (Pan et al., 2016, 2019) and the topographic voltage map (Figure 2), we chose the N400 component with the following 6 electrode sites in the frontal-central areas: FCz, FC1, FC2, Cz, C1, and C2. The ERPs’ waveforms were time-locked to the onset of target stimuli, and the average epoch was 880 ms. The EEG activity during the 80 ms prior to the onset of the target served as the baseline for the analysis of N400. The time window of N400 was 300 ms~400 ms. These time windows were used to compute mean amplitude values. SPSS 16.0 for Windows was used to analyze the behavioral data and ERP data in the present study. All the *p* values of the main and interaction effects were corrected using the Greenhouse-Geisser method.

## Results

### Behavioral Results

#### Flanker Task

**Figure 3:**
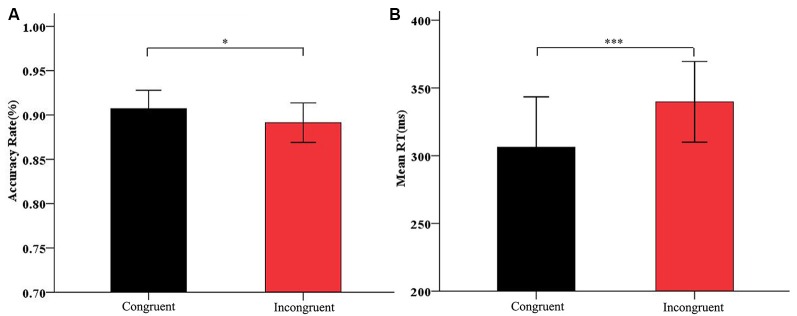
**(A)** Accuracy rate and **(B)** mean RTs for congruent condition and incongruent condition on the Flanker effect. Black bars refer to congruent prime; red bars refer to incongruent prime. Data are represented as the mean and standard deviation. *p< 0.05, ***p < 0.001.

In the analysis of the Flanker task, we analyzed the accuracy rate and the RTs for correct responses on the Flanker task. The accuracy rate for incongruent primes (*M* = 89.12%, *SD* = 6.19) was significantly lower than that for congruent primes (*M* = 90.72%, *SD* = 5.76), *t*_(31)_ = 2.058, *p* < 0.05; (Figure 3A). The RTs for incongruent primes (*M* = 340 ms, *SD* = 82.69) were significantly longer than those for congruent primes (*M* = 306 ms, *SD* = 102.97), *t*_(31)_ = −4.260, *p* < 0.001 (Figure 3B). These results showed that the arrow Flanker task induced a significant conflict effect.

#### Affective Word Task

We analyzed the trials that made correct responses on the affective word tasks after responding correctly to the Flanker task. Hence the accuracy rate of the affective word task would be directly affected by the accuracy rate of the Flanker task. The accuracy rate of the Flanker task had a significant difference between the congruent condition and incongruent condition. As a result, we only analyzed the RTs in the eight conditions by three-factors repeated measures ANOVA: A 2 (reward condition: rewarded vs. unrewarded) × 2 (prime condition: incongruent vs. congruent) × 2 (target valence: positive vs. negative) repeated-measures ANOVA. The analysis showed a significant main effect of reward condition, *F*_(1,31)_ = 15.950, *p* < 0.001, *η*^2^ = 0.340. The main effect of target valence (*F*_(1,31)_ = 6.746, *p* < 0.05, *η*^2^ = 0.179) and prime condition (*F*_(1,31)_ = 22.840, *p* < 0.001, *η*^2^ = 0.424) were significant (Table 1).

**Table 1:** The marginal means for main effects in the analysis of effective word task.

Factor	Condition	*M*	*SD*	*F*	*P*
Reward	Rewarded	609	74	15.950	***
	Unrewarded	640	101		
Congruency	Congruent	619	90	6.746	*
	Incongruent	629	82		
Valence	Positive	605	81	22.840	***
	Negative	644	96		

*p< 0.05, ***p < 0.001.

**Figure 4:**
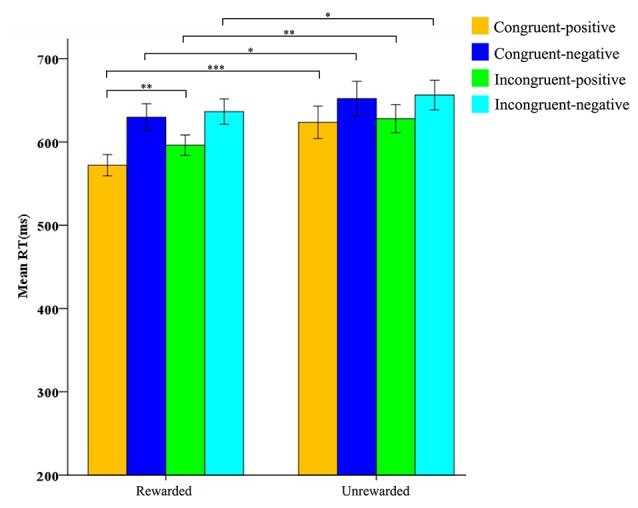
Mean RTs for negative and positive word judgments after congruent and incongruent primes under-rewarded condition and unrewarded condition. Orange bars refer to congruent-positive condition; dark blue bars refer to congruent-negative condition; green bars refer to incongruent-positive condition; light blue bars refer to incongruent-negative condition. The four bars on the left refer to the rewarded (R) condition; the four bars on the right refer to the unrewarded (U) condition. Standard errors are represented. *p< 0.05, ** *p* < 0.01, ***p < 0.001.

Furthermore, the reward condition interacted with prime condition (*F*_(1,31)_ = 4.798, *p* < 0.01, *η*^2^ = 0.028), and target valence (*F*_(1,31)_ = 9.430, *p* < 0.01, *η*^2^ = 0.233). There was a significant interaction among the reward condition, prime condition, and target valance, *F*_(1,31)_ = 5.184, *p* < 0.05, *η*^2^ = 0.143. After studying the interaction further by the simple effect analysis, we found that in the rewarded condition the RTs for positive targets after congruent primes (*M* = 572 ms, *SD* = 72) were significantly shorter than those after incongruent primes (*M* = 596 ms, *SD* = 70), *F*_(1,31)_ = 10.147, *p* < 0.01, *η*^2^ = 0.247. In the unrewarded condition, there was no difference between the RTs for positive targets after congruent primes (*M* = 624 ms, *SD* = 110) and after incongruent primes (*M* = 628 ms, *SD* = 95), *F*_(1,31)_ = 1.415, *p* > 0.05 (Table 2). Finally, we found that the RTs for the four conditions in the rewarded condition were all significantly shorter than those in the unrewarded condition (Figure 4). Other main effects and interactions were non-significant (all *p* > 0.05).

**Table 2:** Simple effect analysis performed on the interaction among the reward condition, prime congruency, and target valence.

Reward	Valence	Congruency	*M*	*SD*	*F*	*P*
Rewarded	Positive	Congruent	572	72	10.147	***
		Incongruent	596	70		
	Negative	Congruent	630	91	1.415	
		Incongruent	636	86		
Unrewarded	Positive	Congruent	624	110	0.343	
		Incongruent	628	95		
	Negative	Congruent	652	117	0.438	
		Incongruent	656	101		

***p < 0.001.

The results of behavioral data showed that the Flanker task induced a significant conflict effect; conflict had no effect on the subsequent processing at the conflict resolution stage, and reward promoted the behavioral performance of participants.

### ERP Waveform Analysis

#### The N2

**Figure 5:**
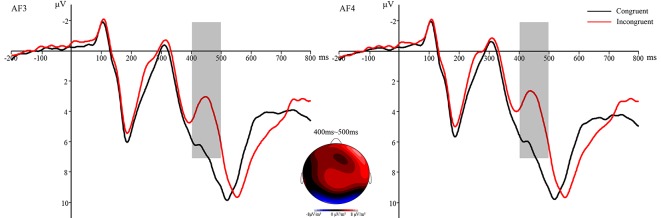
The N2 waveforms were time-locked to the onset of flanker primes—the average N2 at AF3 and AF4 for congruent condition and incongruent condition. Black lines refer to congruent prime; red lines refer to incongruent prime. Topographic voltage map of mean amplitude differences for the conflict effect at 400–500 ms (Congruent minus Incongruent).

The N2 component was analyzed by two-factor repeated measures ANOVA: 5 (electrode sites) × 2 (prime condition: incongruent vs. congruent). The main effect of electrode sites was significant, *F*_(4,124)_ = 8.946, *p* < 0.001, *η*^2^ = 0.224. The main effect of prime condition did interact with the electrode sites, *F*_(4,124)_ = 3.807, *p* < 0.05, *η*^2^ = 0.109. More importantly, the main effect of prime condition was significant, *F*_(1,31)_ = 39.404, *p* < 0.001, *η*^2^ = 0.560, with the N2 amplitudes for incongruent primes (*M* = 3.847 μV, *SD* = 4.15) more negative than those for congruent primes (*M* = 6.879 μV, *SD* = 4.30), showing a significant conflict effect (Figure 5). Other main effects and interactions were non-significant (all *p* > 0.05).

#### The N400

We analyzed the N400 component for correct responses on the affective word tasks after responding correctly to the Flanker task by four-factors repeated measures ANOVA: 6 (electrode sites) × 2 (reward condition: rewarded vs. unrewarded) × 2 (prime condition: incongruent vs. congruent) × 2 (target valence: positive vs. negative). The main effect of electrode sites was significant, *F*_(5,155)_ = 47.502, *p* < 0.001, *η*^2^ = 0.605. The main effect of reward condition was significant, *F*_(1,31)_ = 4.980, *p* < 0.05, *η*^2^ = 0.138. The main effect of target valence was significant, *F*_(1,31)_ = 18.487, *p* < 0.001, *η*^2^ = 0.374. Electrode sites interacted with prime condition, *F*_(5,155)_ = 3.666, *p* < 0.05, *η*^2^ = 0.106. Electrode sites also had a significant interaction with target valence, *F*_(5,155)_ = 8.692, *p* < 0.001, *η*^2^ = 0.219. Moreover, the reward interacted with prime condition, *F*_(1,31)_ = 10.965, *p* < 0.01, *η*^2^ = 0.261.

**Figure 6:**
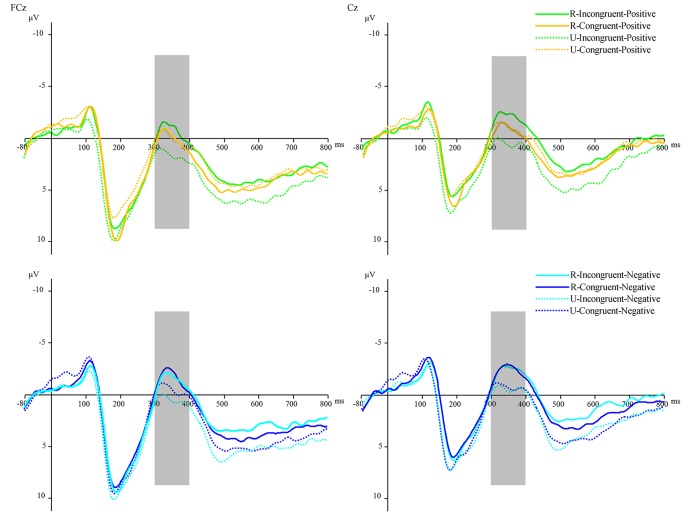
The N400 waveforms were time-locked to the onset of target words—the average N400 at FCz and Cz for all conditions. Orange lines refer to congruent-positive condition; green lines refer to incongruent-positive condition; dark blue lines refer to congruent-negative condition; light blue lines refer to incongruent-negative condition. The solid lines refer to the rewarded condition; the dotted lines refer to the unrewarded condition.

More importantly, there was a significant interaction among reward condition, prime condition, and target valence, *F*_(1,31)_ = 5.004, *p* < 0.05, *η*^2^ = 0.139. After studying the interaction further by the simple effect analysis, in the rewarded condition, we found that the N400 amplitudes for positive targets after incongruent primes (*M* = −0.89 μV, *SD* = 4.65) were significantly more negative than those after congruent primes (*M* = 0.12 μV, *SD* = 4.45), *F*_(1,31)_ = 5.935, *p* < 0.05, *η*^2^ = 0.161, which indicated a significant positive priming effect. While in the unrewarded condition, the N400 amplitudes for positive targets after incongruent primes (*M* = 1.22 μV, *SD* = 4.91) were significantly more positive than those after congruent primes (*M* = 0.12 μV, *SD* = 5.08), *F*_(1,31)_ = 5.837, *p* < 0.05, *η*^2^ = 0.158, which indicated that conflict resolution hindered the processing of positive stimuli. Additionally, no difference was found between the N400 amplitudes for negative targets after congruent primes and the N400 amplitudes for negative targets after incongruent primes in both rewarded and unrewarded conditions (Figure 6).

Moreover, we found that the N400 amplitudes for positive targets after incongruent primes in the rewarded condition (*M* = −0.89 μV, *SD* = 4.65) were significantly more negative than those in the unrewarded condition (*M* = 1.22 μV, *SD* = 4.91), *F*_(1,31)_ = 9.112, *p* < 0.01, *η*^2^ = 0.227; and the N400 amplitudes for negative targets after incongruent primes in the rewarded condition (*M* = −1.24 μV, *SD* = 4.48) were significantly more negative than those in the unrewarded condition (*M* = 0.44 μV, *SD* = 4.51), *F*_(1,31)_ = 8.038, *p* < 0.01, *η*^2^ = 0.206.

The results of EEG data showed that the Flanker task induced a significant conflict effect; Cognitive conflict-induced affective priming effect during the conflict resolution stage, and reward modulated the affective priming effect.

## Discussion

To further explore the role of reward in the affective priming effect, we used ERPs and chose a Flanker task as prime stimulus and the affective words (positive/negative) as target stimuli. Consistent with our hypothesis, we found that the affective priming effect was modulated by the reward. There were two main results: First, the cognitive conflict could induce a positive priming effect during the resolution stage. Second, reward modulated the positive priming effect.

Different from previous studies (e.g., Pan et al., 2016), in order to ensure that the conflict was resolved, participants were asked to respond to the Flanker task first. If they responded correctly, they would have a 50% probability of gaining a reward. In case participants might realize the fixed probability of the presentation of the reward, we blurred the situation in which the rewarded trial would appear. Participants were told that the “quality” of their response to the Flanker task determined whether they would be rewarded or unrewarded. The “quality” of response was a relatively vague concept, and no participant was aware that the reward had a fixed probability. Although we set the probability of reward at 50%, the accuracy of the Flanker task was approximately 90% (incongruent primes: 89.12%; congruent primes: 90.72%); thus, the probability that the participants could obtain a reward was about 45%. We aimed to explore how the reward effect induced by the conflict resolution modulated the subsequent emotion processing. Thus, we set the reward between the primes and the targets rather than before the primes.

It should be noted that the flanker task employed in the present study deviated in critical aspects from a typical flanker task. Specifically, the task was to compare targets and distractors and to indicate whether they do match or not. The named task might raise a question that the named flanker task may be a match-mismatch decision task other than a congruency task. In other words, it might not give rise to cognitive conflict at all. However, we chose and found the N2 component in the present. It was not an essential component analyzed in decision-making studies (Boudreau et al., 2009; Euser et al., 2011), but a critical component analyzed in the studies of cognitive conflict (Schirmer and Kotz, 2003; Zhang et al., 2006; Steinbeis and Koelsch, 2011). Furthermore, the reward was set after the response to the Flanker task but not before the Flanker task; thus, the N2 found in the present could not be the FRN. For the N2 component, we found that the N2 amplitudes for incongruent primes were more negative than those for congruent primes, which showed a significant conflict effect. More importantly, the enhanced N2 amplitudes for incongruent trials reflected higher ACC activation (Botvinick et al., 1999). There is no doubt that the Flanker task successfully induced cognitive conflict.

Some studies (Mulert et al., 2005; Botvinick et al., 2009) found that ACC played a crucial role in allocating cognitive effort exertion in the complex cognitive process, such as working memory (Engström et al., 2013) and decision making (Hauber and Sommer, 2009). As incongruent trials are harder than congruent ones, it is plausible to think that incongruent trials require higher cognitive effort, eliciting higher ACC activation. Recently, EVC theory (Shenhav et al., 2016; Silvetti et al., 2018) makes the ACC role in cognitive effort exertion clearer. Incongruent trials, relative to congruent trials, have higher error probability (lower reward expectation). The amount of control that must be invested to respond correctly to incongruent trials are more than congruent trials. ACC integrated the information above, thereby licensing the higher cognitive effort for incongruent trials.

For the priming effect, the behavioral results were not wholly consistent with those of ERP results. For behavioral results, we found no significant affective priming effect in the rewarded condition and unrewarded condition. We believed that the positive effect induced by conflict resolution might be covered by the positive effect induced by the external rewards because we found that the RTs for the four conditions (i.e., incongruent-positive, incongruent-negative, congruent-positive, and congruent-negative) in the rewarded condition were all significantly shorter than those in the unrewarded condition. This affective effect of conflict resolution should be found in the ERP results for that ERPs could reveal potential processing, while the RTs only reflected the result of the process.

The N400 component (Pan et al., 2016, 2019; Saunders et al., 2017; Pauligk et al., 2019) was chosen to analyze the affective effect of flanker primes on the evaluation of target words. Just as we expected, in the rewarded condition, the N400 amplitudes for positive targets after incongruent primes were significantly more negative than those after congruent primes, which indicated a significant positive priming effect. While in the unrewarded condition, the N400 amplitudes for positive targets after incongruent primes were significantly more positive than those after congruent primes, which indicated that conflicts hinder the process of positive stimuli. In the present study, we tried to set an external reward (reward) and an intrinsic reward (unrewarded). However, with the apparent distinction between the rewarded condition and the unrewarded condition, the intrinsic reward might have been covered and even reverse. Although we only analyzed the correct trails and without setting any punishment, the same feedback stimulus was used to indicate a slow response or incorrect response. The unrewarded trials appeared much more like intrinsic loss or punishment rather than intrinsic reward. Hence participants might experience a negative feeling in the unrewarded condition, which led to a reversal. Further studies are needed to investigate the affective priming effect of cognitive conflicts after an error response or negative feedback. In summary, the N400 results showed that a reward modulated the positive priming effect.

In order to prevent the priming effect from disappearing, the time interval between the resolution of the prime and the presentation of the target could not be long enough to make the baseline stable, and these unstable baselines might result in the unreliability of the data process. To mitigate the influence of a possible offset potential, the pre-stimulus baseline period (80 ms) is a little shorter than is typically recommended. Moreover, a statistical comparison of the baseline period might alleviate this concern. After analyzing the mean amplitudes for baselines (−80 ms~0 ms) in the eight conditions by a four factors repeated-measures ANOVA: 6 (electrode sites) × 2 (reward condition: rewarded vs. unrewarded) × 2 (prime condition: incongruent vs. congruent) × 2 (target valence: positive vs. negative), we found that the interaction among reward condition, prime condition, and target valence was not significant, *F*_(1,31)_ = 3.668, *p* > 0.05. To some extent, we could conclude that our ERP results were reliable.

According to the RVPM, once the congruency task was successfully resolved, it would generate a reward signal even where there is no external reinforcement (Silvetti et al., 2014). We tried to set reward with reinforcement (rewarded trails) and no reinforcement (unrewarded trails). In behavior results, no affective priming effect was found. We suggested that the affective effect of cognitive conflict processing was an internal emotion processing that was covered by the positive effect induced by the external reward in the present study (Inzlicht et al., 2015). Hence in the behavior results, only the reward effect was found. It was fortunate that we used the ERPs, which could reflect the internal processing. In the ERP results, we could conclude that the reward induced by conflict resolution had an emotional effect on subsequent processing and was perceived as a positive priming effect. Moreover, reward modulated the positive effect. Of course, further studies were needed to distinguish the intrinsic reward and external reward so that the modulation of reward on the affective priming effect in cognitive conflict processing is more convincing.

To summarize, cognitive conflict could induce the positive priming effect during the resolution stage, and reward had a moderating effect on the positive priming effect.

## Data Availability Statement

The datasets generated for this study are available on request to the corresponding author.

## Ethics Statement

The studies involving human participants were reviewed and approved by Human Ethics Committee of Nantong University. The patients/participants provided their written informed consent to participate in this study.

## Author Contributions

FP and YO designed and coordinated the study. YO and XZ carried out experiments and data processes. YO drafted the manuscript. FP reviewed the manuscript. All authors gave final approval for publication.

## Conflict of Interest

The authors declare that the research was conducted in the absence of any commercial or financial relationships that could be construed as a potential conflict of interest.
